# The Effects of Hopelessness on Chronic Disease Among African Americans and Caribbean Blacks: Findings from the National Survey of American Life (NSAL)

**DOI:** 10.1007/s10597-019-00536-z

**Published:** 2020-01-02

**Authors:** Michael A. Robinson, Irang Kim, Orion Mowbray, Tiffany Washington

**Affiliations:** 1 School of Social Work, The University of Georgia, 279 Williams St., Athens, GA 30602 USA; 2 Present Address: School of Social Work, University of Maryland, Baltimore, 525 W. Redwood Street, Baltimore, MD 21201 USA

**Keywords:** African Americans mental health, Depression, Hopelessness, Caribbean Blacks mental health, National Survey of American Life, Chronic disease

## Abstract

This paper examines the relationship between hopelessness on chronic disease in a national sample of African Americans (3570) and Caribbean Blacks (1438) Using the National Survey of American Life. A multivariate negative binomial regression examined whether chronic disease is associated with hopelessness, controlling for sociodemographic characteristics. Overall, 13.14% of the sample reported they were highly hopeless, and 31.5% indicated they were moderately hopeless. About 19% of respondents experienced chronic disease. Bivariate associations showed that those who have ever had chronic disease significantly differed from those who did not in regard to age, gender and spirituality. Multivariate results showed that respondents who ever have had chronic disease reported significantly higher hopelessness scores than those with no chronic disease. The study findings contribute to the current body of literature by supporting findings from smaller studies on the relationship between depression and hopelessness in African Americans and Caribbean Blacks.

## Introduction

The African American and Caribbean Black population in the U.S. is growing. The African Americans population will increase from 12.3% of the U.S. population to 17.9% by 2060 (U.S. Census 2015). Likewise, the population of Black immigrants in the U.S has quadrupled since 1980 to 3.8 million with approximately 50% coming from the Caribbean (Anderson 2015). With the growing population of African Americans and Caribbean Blacks, research is needed to explore the health of these populations, as it is widely known that Blacks in the U.S. disproportionately experience poorer health outcomes than non-Hispanic Whites (Everson-Rose and Lewis 2005). Currently, there are very few national studies that examine the health of these populations (Griffith et al. 2011) and even fewer studies have examined how Blacks cope with chronic disease. Prior research shows that optimism and pessimism affects health outcomes (Peterson et al. 1988) and this study aims to build on previous research by exploring the effects of hopelessness on chronic disease in a national sample of African Americans and Caribbean Blacks in the U.S.

## Chronic Disease

The CDC (2006) defines chronic disease as “noncommunicable illnesses that are prolonged in duration, do not resolve spontaneously, and are rarely cured completely” (p. 2). Chronic diseases are prevalent, co-occur, among the leading causes of death in the U.S., and expensive to treat. Nearly half of all adults in the U.S. live with at least one chronic condition and more than 25% live with more than one chronic condition (Ward et al. 2012; CDC 2016). Moreover, chronic disease account for 7 out of 10 deaths annually, and 86% of all healthcare expenditures are associated with chronic diseases (CDC 2016). The CDC identifies the common causes of chronic disease are lack of physical activity, inadequate nutrition, tobacco use, and excessive consumption of alcohol (CDC 2009b, c, 2016), and urbanization (Yach et al. 2004). Not only are they costly, they result in disability and premature death (CDC 2009b, c).

Disparities in chronic disease are a cause for concern. For instance, the incidence and mortality rates for chronic disease vary by race. Whites account for 46% of chronic disease compared to 37% among Blacks (Anderson and Horvath 2004). Although Whites experience higher levels of chronic diseases among the general United States population (Anderson and Horvath 2004), African Americans disproportionately experience poorer outcomes and are more likely to die prematurely from cardiovascular diseases, such as hypertension, coronary heart disease, myocardial infarction and stroke (Everson-Rose and Lewis 2005), and diabetes (CDC 2009a). Equally concerning are chronic disease-associated age disparities. The prevalence rate for chronic disease increases with age (Gerteis et al. 2014; Anderson and Horvath 2004). Many older adults aged 65 years and older have more than one chronic condition, which suggests the prevalence of multiple chronic diseases in old age (Anderson and Horvath 2004).

## Burden of Chronic Disease

In light of the individual and societal costs of chronic disease, the U.S. government allotted $650 million dollars from the *American Recovery and Investment Act of 2009* in the form of a community health initiative, *Communities Putting Prevention to Work* (Health and Human Services [HHS] 2011). The chronic diseases that have been the focus of prevention efforts in the United States include heart disease, stroke, cancer, diabetes, arthritis, obesity, respiratory diseases, and oral conditions (CDC 2009b, c).

Anderson and Horvath (2004) examined the Medical Expenditure Panel Survey data from 1998. Findings from their research suggest that approximately four out of five health care dollars were spent on chronic diseases. Findings also suggest that individuals with chronic disease were heavy utilizers of home health services, prescriptions drugs, physician visits, and inpatient hospitalizations. For many individuals living with a chronic disease, life is fraught with challenges. Everson-Rose et al. (2005), as well as others (Anda et al. 1993; Brody et al. 2008; Else-Quest et al. 2009; St. Rose and Wilson 2009) have written about the impact of chronic diseases on well-being. Given their pervasiveness, costliness, and intrusiveness on daily life, it is reasonable to assume that living with a chronic disease can threaten a person’s psychological well-being and undermine feelings of hope, especially in Black Americans who already experience race-related discrimination. Chronic disease burden is associated with depressive and stress symptoms (Katon et al. 2007), but little research has examined the relationship between chronic disease and hopelessness in African Americans and Caribbean Blacks. Much of the research literature on hopelessness and chronic diseases focuses on morbidity and mortality from cardiovascular diseases, or among predominantly White samples. This paper examines the relationship between hopelessness on chronic disease in a national sample of African Americans and Caribbean Blacks.

## Hopelessness

Hopelessness is “negative expectancies about oneself and the future” (Everson et al. 1997, p. 1491). There is a significant body of evidence in the research literature that suggests a lack of hope can negatively affect physical and mental health (Anda et al. 1993; Everson et al. 1997; Kuosmanen 2016; Pedersen et al. 2007; Valtonen et al. 2008; Whipple et al. 2009). More specifically, evidence suggests that hopelessness yields a strong and independent influence on cardiovascular disease outcomes in the U. S. and other countries (Everson et al. 1996). Furthermore, Blacks are more likely to demonstrate depressed affect and hopelessness more than Whites (Anda et al. 1993). Individuals with either depressed affect or hopelessness are at an increased risk for fatal and non-fatal IHD independent of IHD risks. Moreover, high levels of hopelessness accelerate atherosclerosis as demonstrated by intima-media thickening (IMT), plaque height, and surface roughness of the common carotid artery (Everson et al. 1997).

Similarly, among patients who received paclitaxel-eluting stents, depressive symptoms, such as hopelessness, were associated with adverse clinical outcomes, and the incidence of death/non-fatal myocardial infarction at 2 years post-procedure increased threefold among participants with high levels of hopelessness (Pederson et al. 2007). Finally, in a cross-sectional study that examined the associations of hopelessness and depressive symptoms among middle-aged White and Black female patients with carotid artery intima-media thickness (IMT), high levels of hopelessness were associated with greater IMT (Whipple et al. 2009), which is consistent with findings from other studies (Everson et al. 1997). The findings were particularly clinically significant due to the relationship between hopelessness and mean IMT. As the level of hopelessness increased, the level of mean IMT also increased and as the level of hopelessness decreased, as did the level of mean IMT.

Associations between hopelessness and metabolic syndrome are also established. For instance, hopelessness was strongly associated with metabolic syndrome, independent of depressive symptoms and risk factors for cardiovascular diseases (Valtonen et al. 2008). High levels of hopelessness have a twofold greater risk for metabolic syndrome compared to low levels of hopelessness.

The clinical and empirical evidence of the negative effects of hopelessness among patients with chronic health conditions, such as cardiovascular diseases, has been well established in the research literature. Much research has been conducted on the negative effects of hopelessness on health; however, findings from this literature review suggest opportunities for more research on the complex associations of hopelessness and health. For example, research suggests that hopelessness (Everson et al. 1996; Pedersen et al. 2007; Valtonen et al. 2008) can negatively affect health; likewise, research also suggests that chronic disease, such as stroke (Fung et al. 2006), can result in an increase of hopelessness. Most of the current literature on hopelessness and chronic disease does not have a primary focus on African Americans; therefore, the current study proposes to examine the relationship between hopelessness and chronic disease on a national sample of African Americans and Caribbean Blacks. This study’s hypothesis is that African American and Caribbean Blacks living with a chronic disease will be more likely to experience hopelessness than those living without a chronic disease.

## Methods

### Sample

The present study is a secondary analysis of the National Survey of American Life (NSAL), which conducted 6082 interviews with adults 18 years and older that were collected between 2001 through 2003 (Jackson et al. 2004; Pennell et al. 2004). Eighty-six percent of the interviews were face to face and 14% were telephone interviews with participants who self-identified in the following three categories: African American, Caribbean Black, and non-Latino White. However, for the purpose of the present study, the researchers will examine the African American (n = 3570) and Caribbean Black (n = 1438) populations (total n = 5008) because they comprise the largest population of Blacks in the U.S. The NSAL is the largest dataset to date on mental health and African Americans and was collected from a representative sample of the US adult population. The NSAL project is supported by the National Institutes of Health and the National Institute of Mental Health, and receives supplemental support from the Office of Behavioral and Social Sciences Research.

### Measures

#### Chronic Disease

A dichotomous variable indicated the presence of chronic disease. The variable measured whether the participants have ever had one of four chronic diseases; heart disease, cancer, stroke or diabetes. The selection of specific chronic disease was based on Centers for Disease Control guidelines (Hootman et al. 2009). Chronic disease was assessed by asking the questions, (1) Has a professional ever said you have heart trouble? (2) Has a professional ever said you have cancer? (3) Has a professional ever said you had a stroke? (4) Has a professional ever said you have diabetes? Participants who said yes to at least one of these questions was considered to have a chronic disease.

#### Hopelessness

Hopelessness was measured using the sum score of the Everson Hopelessness Scale (Everson 1995), which consisted of two items (1) I sometimes think I am no good, and (2) It is impossible to reach my goals. These items were summed to create the Hopelessness Scale. A hopelessness scale score of 3, 4, or 5 indicate moderate hopelessness and scores of 6, 7, or 8 indicate high hopelessness (Everson et al. 1996). Range of hopelessness scores were from 0 to 8 (Mean = 3.25, SD = 1.76). The correlation coefficient for the two items was 0.63.

#### Sociodemographics

Participants also reported their age, gender, income, employment status, employment status and spirituality. Work status was measured with three categorical variables: employed, unemployed, and not in labor force. Spirituality was assessed along a four-point response scale (1 = not spiritual at all, 2 = not too spiritual, 3 = fairly spiritual, 4 = very spiritual. All sociodemographic variables were selected based on the literature review that showed associations with hopelessness.

### Analytic Strategy

All analyses were completed in STATA Version 12 (Statacorp 2011). Bivariate associations examined differences in hopelessness and sociodemographics between those who have ever had a chronic disease and those who have not. Because hopelessness is a based on a count measure, and is not a true continuous measure, a negative binomial regression examined whether chronic disease is associated with hopelessness, controlling for sociodemographic characteristics. Analysis incorporated the survey weighting design provided in NSAL.

## Results

### Sample Characteristics and Bivariate Associations

Overall, 13.14% of the entire sample reported they were highly hopeless, and 31.5% indicated they were moderately hopeless. Table 1 shows a summary of sample characteristics and bivariate associations with hopelessness. About 19% of respondents experienced chronic disease. Age of participants in the study ranged from 18 to 94 years (Mean = 42.54). About 24% of respondents reported less than 11 years of education, 36% completed 12 years’ education, 24% completed 13–15 years, and 17% reported more than 16 years. About 68% of respondents were employed and the largest percentage of respondents reported an annual income between $35,000 and $69,999. The mean response for spirituality (3.31) corresponded with the “fairly spiritual” designation.

**Table 1:** Sample descriptive and bivariate associations with chronic disease

Characteristics	Overall % N = 5008 % (M)	Chronic disease^a^ N = 942 % (M)	No chronic disease N = 4066 % (M)	χ^2^ (*F*)
Race				19.66**
Caribbean Black	28.71	14.95	85.05	
African American	71.29	20.36	79.64	
Age				
18 to 24	14.00	4.14	16.28	546.45**
25 to 34	21.59	10.93	24.05	
35 to 54	42.29	35.46	43.88	
55 and over	22.12	49.47	15.79	
Gender				6.43*
Male	36.60	33.01	37.43	
Female	63.40	66.99	62.57	
Education				75.90**
0–11 years	23.78	34.50	21.30	
12 years	35.64	31.95	36.50	
13–15 years	23.86	18.68	25.06	
Greater than 16 years	16.71	14.86	17.14	
Income^b^				95.77**
0–9999	15.46	22.51	13.82	
10,000–19,999	22.32	28.98	20.78	
20,000–34,999	23.80	17.94	25.16	
35,000–69,999	27.68	21.76	29.05	
70,000 or more	10.74	8.81	11.19	
Work				362.33**
Employed	68.05	46.50	73.05	
Unemployed	9.98	8.49	10.33	
Not in labor force	21.97	45.01	16.62	
Spirituality^c^	(3.31)	(3.44)	(3.28)	36.30**

Numbers in column headings represent unweighted values. All analyses incorporate the NSAL sample weighting strategy

*p < 0.05, **p < 0.01

^a^Measured as the presence of heart disease, cancer, stroke or diabetes

^b^Measured as annual household income

^c^4 point scale 1 = not spiritual at all, 2 = not too spiritual, 3 = fairly spiritual, 4 = very spiritual

Bivariate associations showed that those who have ever had chronic disease (M = 3.60) reported significantly higher hopelessness than those who do not have chronic disease (M = 3.13), *F* (1, 54) = 41.26, p < 0.01. Bivariate analyses also showed that hopelessness were significantly affected by years of educational attainment (*F* (3, 52) = 60.37, p < 0.01), income (*F*(4, 51) = 27.03, p < 0.01), and work status (*F* (2, 53) = 20.35, p < 0.01). Last, spirituality was negatively associated with hopelessness (*F* (1, 54) = 20.00, p < 0.01).

When examining differences between African American and Caribbean Black individuals, African American individuals (20.36%) were significantly more likely than Caribbean Black individuals (14.95%) to have chronic disease (χ^2^ (1, N = 5008) = 19.66, p < 0.01). In addition, Caribbean Blacks reported higher education (χ^2^ (3, N = 5008) = 130.93, p < 0.01) and higher annual income (χ^2^ (3, N = 5008) = 133.27, p < 0.01) compared to African Americans.

### Multivariate Model Predicting Hopelessness

Results from negative binomial regression were presented in Table 2. This table displays the incidence rate ratios and 95% confidence interval for each independent variable. IRRs are conceptualized as a standardized coefficient, enabling comparisons across independent variables, similar to standardized coefficients in linear regression. Model results showed that respondents who ever have had chronic disease reported significantly higher hopelessness scores than those who have not had the chronic disease (IRR = 1.11, *p* < 0.01). African Americans were less likely to be hopelessness compared to Caribbean Black (IRR = 0.931, *p* < 0.05). Furthermore, those with higher education, higher income, and higher spirituality reported significantly lower hopelessness scores. Education and income were also significantly associated with hopelessness such that higher education and higher income were associated with lower hopelessness scores. Additionally, higher levels of spirituality were associated with lower hopelessness scores (IRR = 0.943, p < 0.01).

**Table 2:** Negative binomial regression model of hopelessness

Characteristics	Hopelessness^a^ (N = 5008)	
	IRR	95% CI
Chronic illness	1.11**	[1.06, 1.16]
Race (African American)	0.931*	[0.869, 0.998]
Gender (Male)	1.00	[0.956, 1.05]
Age		
18 to 24	–	–
25 to 34	0.998	[0.925, 1.08]
35 to 54	1.03	[0.953, 1.11]
55 and over	0.967	[0.892, 1.05]
Education		
0–11 years	–	–
12 years	0.883**	[0.837, 0.931]
13–15 years	0.814**	[0.771, 0.859]
Greater than 16 years	0.779**	[0.736, 0.826]
Income^b^		
0–9999	–	–
10,000–19,999	0.931*	[0.867, 0.999]
20,000–34,999	0.858**	[0.796, 0.925]
35,000–69,999	0.837**	[0.782, 0.896]
70,000 or more	0.829**	[0.751, 0.917]
Work		
Employed	–	–
Unemployed	1.07	[0.997, 1.16]
Not in labor force	1.07*	[1.00, 1.14]
Spirituality^c^	0.943**	[0.918, 0.969]

Numbers in column headings represent unweighted values. All analyses incorporate the NSAL sample weighting strategy

*p < 0.05, **p < 0.01

^a^Measured as the presence of heart disease, cancer, stroke or diabetes

^b^Measured as annual household income

^c^4 point scale 1 = Not spiritual at all, 2 = Not too spiritual, 3 = Fairly spiritual, 4 = Very spiritual

## Discussion

The current study was an examination of the relationship between hopelessness and chronic disease in a national sample of African Americans and Caribbean Blacks. Findings in this study are consistent with research findings that hopelessness can negatively affect health. However, this study’s contribution is the primary focus on African Americans and Caribbean Blacks, which is important given these populations experience a disproportionate burden of chronic disease, such as diabetes and stroke, in comparison to other racial and ethnic groups (CDC 2016). This study’s main hypothesis was supported: respondents with chronic disease had 1.11 times the risk of experiencing hopelessness than those without. This finding is not surprising given the intrusiveness of chronic disease in the lives of individuals and family systems (Rolland 1994). Also, this study found discrimination was positively associated with hopelessness. Previous studies have elucidated how experiences with discrimination over the life course can negatively impact health and mental health conditions (Chae et al. 2010; Mereish et al. 2016). This discovery is worthy of further investigation as it could point to additional evidence that discrimination and mental health are linked.

In addition, this study contributes to existing knowledge by identifying socioeconomic mechanisms by which hopelessness is associated with chronic disease. In this study, education, employment, and income influenced feelings of hopelessness. Future examinations may consider how socioeconomic status contributes to other psychological factors in the context of chronic disease, such as depression. Previous research highlights income and unemployment as having a greater odds of having depression in African American men (Hudson et al. 2011); thus, an examination of the joint contribution of socioeconomic factors, depression, and hopelessness among African American and Caribbean Blacks could be a first step toward generating a psychosocial profile linked to chronic disease. Also, given the growing evidence that discrimination relates to conditions that disproportionately affect African Americans such as kidney and heart disease (Beydoun et al. 2017; Chae et al. 2012), there is an opportunity to better understand the roles of hopelessness and discrimination in specific chronic conditions that are pervasive and costly.

In this study, spirituality showed an opposite direction of association with hopelessness, compared to the direction of association for chronic disease and hopelessness (e.g., those with higher levels of spirituality had lower levels of hopelessness). This finding is not surprising, as previous studies have found that in comparison to Whites, Black men and women report higher levels of spirituality (Taylor et al. 2009), and faith-based organizations (i.e., Black churches) have long served as cornerstone institutions and sources of support in Black communities (Lincoln and Mamiya 1990). Future modeling is needed to determine if spirituality can mediate or moderate the relationship between chronic disease and hopelessness. Explicating the mediation or moderation potential of spirituality may point to directions for practice interventions. Lastly and equally important, findings suggest opportunity for studies that incorporate longitudinal designs and larger samples of African Americans and other minorities.

This study’s findings contribute to knowledge about factors that relate to the health of African American and Caribbean Blacks; however, its cross-sectional design limited the ability to examine the effect of hopelessness and other psychosocial factors on chronic disease. Hence, temporal order among the variables could not be established, and as suggested above, longitudinal designs are needed. In conclusion, chronic disease is the leading cause of death in the U.S. and warrants more research as one half of all adults live with at least one chronic disease. A better understanding and awareness may help public health professionals and clinicians to develop culturally-sensitive screenings if cultural competency is to be broadly adopted in health systems. Health systems can better serve African American and Caribbean Blacks by incorporating their values and preferences for treatment into health and mental health services, which could lead to better outcomes (Mowbray et al. 2018). That is, providers’ efforts to incorporate culture into the design and delivery of services may positively impact hopelessness, a hypothesis worth testing in a future study. Lastly, culturally sensitive health professionals who understand the role of hopelessness or any other comorbid psychological conditions in the treatment of chronic diseases are also needed, as the current research indicates that there is a relationship, and the relationship varies by race and ethnicity.
